# Prebiotics: Definition, Types, Sources, Mechanisms, and Clinical Applications

**DOI:** 10.3390/foods8030092

**Published:** 2019-03-09

**Authors:** Dorna Davani-Davari, Manica Negahdaripour, Iman Karimzadeh, Mostafa Seifan, Milad Mohkam, Seyed Jalil Masoumi, Aydin Berenjian, Younes Ghasemi

**Affiliations:** 1 Pharmaceutical Biotechnology Incubator, School of Pharmacy, Shiraz University of Medical Sciences, Shiraz 71348, Iran; d.davani.d@gmail.com; 2 Department of Pharmaceutical Biotechnology, School of Pharmacy, Shiraz University of Medical Sciences, Shiraz 71348, Iran; Manica.Negahdaripour@gmail.com; 3 Pharmaceutical Sciences Research Center, Shiraz University of Medical Sciences, Shiraz 71348, Iran; 4 Department of Clinical Pharmacy, School of Pharmacy, Shiraz University of Medical Sciences, Shiraz 71348, Iran; karimzadehiman@yahoo.com; 5 Faculty of Science and Engineering, University of Waikato, Hamilton 3216, New Zealand; Aydin.berenjian@waikato.ac.nz; 6 Biotechnology Research Center, Shiraz University of Medical Sciences, Shiraz 71348, Iran; Milad.Mohkam47@yahoo.com; 7 Nutrition Research Center, Department of Clinical Nutrition, School of Nutrition and Food Sciences, Shiraz University of Medical Sciences, Shiraz 71348, Iran; J.masoumi74@gmail.com; 8 Department of Medical Biotechnology, School of Advanced Medical Sciences and Technologies, Shiraz University of Medical Sciences, Shiraz 71348, Iran

**Keywords:** prebiotics, gut microbiota, short-chain fatty acids, fructo-oligosaccharides, galacto-oligosaccharides

## Abstract

Prebiotics are a group of nutrients that are degraded by gut microbiota. Their relationship with human overall health has been an area of increasing interest in recent years. They can feed the intestinal microbiota, and their degradation products are short-chain fatty acids that are released into blood circulation, consequently, affecting not only the gastrointestinal tracts but also other distant organs. Fructo-oligosaccharides and galacto-oligosaccharides are the two important groups of prebiotics with beneficial effects on human health. Since low quantities of fructo-oligosaccharides and galacto-oligosaccharides naturally exist in foods, scientists are attempting to produce prebiotics on an industrial scale. Considering the health benefits of prebiotics and their safety, as well as their production and storage advantages compared to probiotics, they seem to be fascinating candidates for promoting human health condition as a replacement or in association with probiotics. This review discusses different aspects of prebiotics, including their crucial role in human well-being.

## 1. Introduction

Various types of microorganisms, known as gut microbiota, are inhabitants of the human gastrointestinal tract. It has been reported that there are 10^10^–10^12^ live microorganisms per gram in the human colon [1]. The resident microbial groups in the stomach, small, and large intestine are crucial for human health. The majority of these microorganisms, which are mostly anaerobes, live in the large intestine [2].

Although some endogenous factors, such as mucin secretions, can affect the microbial balance, human diet is the chief source of energy for their growth. Particularly, non-digestible carbohydrates can highly modify the composition and function of gut microbiota [3]. Beneficial intestinal microbes ferment these non-digestible dietary substances called prebiotics and obtain their survival energy from degrading indigestible binds of prebiotics [4,5]. As a result of this, prebiotics can selectively influence gut microbiota [6,7]. On the other hand, the gut microbiota affects intestinal functions, such as metabolism and integrity of the intestine. Moreover, they can suppress pathogens in healthy individuals through induction of some immunomodulatory molecules with antagonistic effects against pathogens by lactic acid that is produced by *Bifidobacterium* and *Lactobacillus* genera [8,9,10,11].

Various compounds have been tested to determine their function as prebiotics. Fructo-oligosaccharides (FOS), galacto-oligosaccharides (GOS), and trans-galacto-oligosaccharides (TOS) are the most common prebiotics. Fermentation of prebiotics by gut microbiota produces short-chain fatty acids (SCFAs), including lactic acid, butyric acid, and propionic acid. These products can have multiple effects on the body. As an example, propionate affects T helper 2 in the airways and macrophages, as well as dendritic cells in the bone marrows [12,13]. SCFAs decrease the pH of colon [14,15]. Peptidoglycan is another prebiotics fermentation product that can stimulate the innate immune system against pathogenic microorganisms [12,16]. The structure of prebiotics and the bacterial composition of gut determine the fermentation products [14,15]. The effects of prebiotics on human health are mediated through their degradation products by microorganisms. For example, butyrate influences intestinal epithelial development [17]. Since SCFAs can diffuse to blood circulation through enterocytes, prebiotics have the ability to affect not only the gastrointestinal tract but also distant site organs [18].

In this review, we critically elaborate on different aspects of prebiotics, including their definition, types, sources, mechanisms, and clinical applications.

## 2. Definition

The prebiotics concept was introduced for the first time in 1995 by Glenn Gibson and Marcel Roberfroid [4]. Prebiotic was described as “a non-digestible food ingredient that beneficially affects the host by selectively stimulating the growth and/or activity of one or a limited number of bacteria in the colon, and thus improves host health”. This definition was almost unchanged for more than 15 years. According to this definition, only a few compounds of the carbohydrate group, such as short and long chain β-fructans [FOS and inulin], lactulose, and GOS, can be classified as prebiotics. In 2008, the 6th Meeting of the International Scientific Association of Probiotics and Prebiotics (ISAPP) defined “dietary prebiotics” as “a selectively fermented ingredient that results in specific changes in the composition and/or activity of the gastrointestinal microbiota, thus conferring benefit(s) upon host health” [19].

The following criteria are used to classify a compound as a prebiotic: (i) it should be resistant to acidic pH of stomach, cannot be hydrolyzed by mammalian enzymes, and also should not be absorbed in the gastrointestinal tract, (ii) it can be fermented by intestinal microbiota, and (iii) the growth and/or activity of the intestinal bacteria can be selectively stimulated by this compound and this process improves host’s health [19].

Although not all the prebiotics are carbohydrates, the following two criteria can be exploited to distinguish fiber from carbohydrate-derived prebiotics: (i) fibers are carbohydrates with a degree of polymerization (DP) equal or higher than 3 and (ii) endogenous enzymes in the small intestine cannot hydrolyze them. It should be taken into account that the fiber solubility or fermentability is not crucial [20,21].

There are also some revised definitions for prebiotics published in the scientific literature [22]. However, the above-mentioned definition, which was given in 2008, has been accepted in recent years. Despite the absence of a consensus definition, the important part of the original and other definitions is that the consumption of prebiotics is associated with human well-being. The word “selectivity”, or the potency of a prebiotic to stimulate a specific gut microbiota, was another key element of the original definition; however, this concept has been questioned recently [23]. In 2013, Scott et al. [24] reported that the prebiotic effect was enhanced by cross-feeding, defined as the product of one species which can be consumed by another one. This implication raises doubt for utilizing the “selectivity” term in the prebiotics definition. A review on the evolution of prebiotics concept through history can be found in a previous publication [23], and the debate on their definition is still ongoing [25].

## 3. Types of Prebiotics

There are many types of prebiotics. The majority of them are a subset of carbohydrate groups and are mostly oligosaccharide carbohydrates (OSCs). The relevant articles are mainly on OSCs, but there are also some pieces of evidence proving that prebiotics are not only carbohydrates.

### 3.1. Fructans

This category consists of inulin and fructo-oligosaccharide or oligofructose. Their structure is a linear chain of fructose with β(2→1) linkage. They usually have terminal glucose units with β(2→1) linkage. Inulin has DP of up to 60, while the DP of FOS is less than 10 [2].

Previously, some studies implicated that fructans can stimulate lactic acid bacteria selectively. However, over recent years, there are some investigations showing that the chain length of fructans is an important criterion to determine which bacteria can ferment them [26]. Therefore, other bacterial species can also be promoted directly or indirectly by fructans.

### 3.2. Galacto-Oligosaccharides

Galacto-oligosaccharides (GOS), the product of lactose extension, are classified into two subgroups: (i) the GOS with excess galactose at C_3_, C_4_ or C_6_ and (ii) the GOS manufactured from lactose through enzymatic trans-glycosylation. The end product of this reaction is mainly a mixture of tri- to pentasaccharides with galactose in β(1→6), β(1→3), and β(1→4) linkages. This type of GOS is also termed as trans-galacto-oligosaccharides or TOS [19,27].

GOSs can greatly stimulate *Bifidobacteria* and *Lactobacilli*. *Bifidobacteria* in infants have shown high incorporation with GOS. *Enterobacteria*, *Bacteroidetes*, and *Firmicutes* are also stimulated by GOS, but to a lesser extent than *Bifidobacteria* [2].

There are some GOSs derived from lactulose, the isomer of lactose. This lactulose-derived GOSs are also considered as prebiotics [19]. Besides these types of GOS, the other types are based on sucrose extension named raffinose family oligosaccharides (RFO). The effect of RFO on gut microbiota has not been elucidated yet [28,29].

### 3.3. Starch and Glucose-Derived Oligosaccharides

There is a kind of starch that is resistant to the upper gut digestion known as resistant starch (RS). RS can promote health by producing a high level of butyrate; so it has been suggested to be classified as a prebiotic [30]. Various groups of *Firmicutes* show the highest incorporation with a high amount of RS [3]. An in vitro study demonstrated that RS could also be degraded by *Ruminococcus bromii*, and *Bifidobacterium adolescentis*, and also to a lesser extent by *Eubacterium rectale* and *Bacteroides thetaiotaomicron*. However, in the mixed bacterial and fecal incubations, RS degradation is impossible in the absence of *R. bromii* [31].

Polydextrose is a glucose-derived oligosaccharide. It consists of glucan with a lot of branches and glycosidic linkages. There is some evidence that it can stimulate *Bifidobacteria*, but it has not been confirmed yet [32].

### 3.4. Other Oligosaccharides

Some oligosaccharides are originated from a polysaccharide known as pectin. This type of oligosaccharide is called pectic oligosaccharide (POS). They are based on the extension of galacturonic acid (homogalacturonan) or rhamnose (rhamnogalacturonan I). The carboxyl groups may be substituted with methyl esterification, and the structure can be acetylated at C_2_ or C_3_. Various types of sugars (e.g., arabinose, galactose, and xylose) or ferulic acid are linked to the side chains [33]. Their structures vary significantly depending on the sources of POSs [34].

### 3.5. Non-Carbohydrate Oligosaccharides

Although carbohydrates are more likely to meet the criteria of prebiotics definition, there are some compounds that are not classified as carbohydrates but are recommended to be classified as prebiotics, such as cocoa-derived flavanols. In vivo and in vitro experiments demonstrate that flavanols can stimulate lactic acid bacteria [35].

## 4. Production of Prebiotics

**Figure 1:**
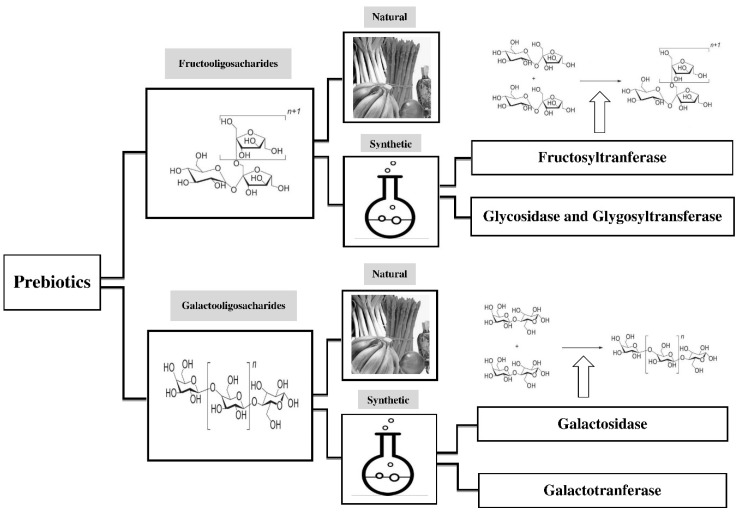
Sources and production of major prebiotics, including fructo-oligosaccharides (FOS) and galacto-oligosaccharides (GOS). Prebiotics exist in human diets in small concentration. Since they have crucial roles in health maintenance, they are manufactured on industrial large scales.

Prebiotics play an important role in human health. They naturally exist in different dietary food products, including asparagus, sugar beet, garlic, chicory, onion, Jerusalem artichoke, wheat, honey, banana, barley, tomato, rye, soybean, human’s and cow’s milk, peas, beans, etc., and recently, seaweeds and microalgae [36]. Because of their low concentration in foods, they are manufactured on industrial large scales. Some of the prebiotics are produced by using lactose, sucrose, and starch as raw material [37,38]. Since most prebiotics are classified as GOS and FOS regarding industrial scale (Figure 1), there are many relevant studies on their production.

### 4.1. FOS

FOS exists in about 36,000 plants [39]; however, the concentration of FOS in these sources is not enough to have prebiotics effects. Therefore, FOS should be synthesized. There are various FOS production methods, which have been explained by several authors [40,41]. FOS can be synthesized chemically by using glycosidase and glycosyl-transferase [42]. The compounds that are used in these reactions are hazardous and costly, and the concentration of the end product (FOS) is very low. Thus, it cannot be produced on an industrial scale [43]. Fructosyl-transferase (FTase) is a key enzyme in producing FOS. FTase produces FOS from sucrose by transferring one to three molecules of fructose. Several microorganisms have FTase, such as *Fusarium* sp., *Aspergillus* sp., *Aureobasidium* sp., *Penicillium* sp., *Arthrobacter* sp., *Zymomonas mobilis*, *Bacillus macerans*, *Candida, Kluyveromyces*, and *Saccharomyces cerevisia*e [41,44,45,46,47,48]. Among these microorganisms, *Aspergillus niger* and *Aureobasidium pullulans* are mostly used in the industry [49].

For FOS production, the whole cell of a microorganism or free enzyme can be used [40,45,50]. There are different factors that can affect the concentration of produced FOS. The maximum amount of FOS produced by FTases depends on the initial concentration of sucrose (theoretically around 55–60%). Glucose, which is a co-product of fermentation, inhibits trans-glycosylation [40,51]. Therefore, removing glucose and sucrose residues is a critical step to achieving higher yields of FOS fermentation. Some scientists claimed to utilize glucose oxidase and β-fructofuranosidase to enhance the yield of FOS production [41,51,52]. β-fructofuranosidase is capable of converting sucrose to FOS. The glucose produced during FOS fermentation is converted to gluconic acid by glucose oxidase. Unlike glucose, gluconic acid is able to be removed by ion-exchange resins or by coagulation with calcium carbonate (CaCO_3_) [52]. Thus, the utilization of both enzymes increases the yield of FOS formation up to 98% [53]. β-fructofuranosidase and glucose oxidase can be derived from *Apostichopus japonicus* and *A. niger*, respectively [54]. Glucose can be separated from FOS through nanofiltration methods. This process increases FOS production by up to 90% [55].

*S. cerevisiae* and *Zymomonas mobilis* are able to eliminate small saccharides, such as glucose, fructose, and sucrose, by converting saccharides to carbon dioxide and ethanol. *S. cerevisiae* cannot ferment oligosaccharides with four or more monosaccharide units. Sorbitol and FOS are also produced in small amounts during fermentation of sucrose by *Z. mobilis* [56,57,58,59].

### 4.2. GOS

GOSs were first chemically synthesized by nucleophilic and electrophilic displacement, but this method is currently deemed to be uneconomical at the industrial scale [60,61]. The key enzymes for GOS formation are galactosyl-transferase and galactosidase. Galactosyl-transferase is a stereoselective enzyme that can produce GOS in high quantities [61]. Nevertheless, the bio-catalysis of GOS via galactosyl-transferase is so costly, because this reaction needs nucleotide sugars as a donor. There are some approaches to decrease the cost of this reaction, such as globotriose production [60,62] or using human milk oligosaccharides [63,64].

Formation of GOS by means of galactosidase is much cheaper than galactosyl-transferases. However, galactosidase produces GOS in lower quantities, and this enzyme is less stereospecific than galactosyl-transferase. The amount of GOS produced by galactosidase can be improved in different ways: (i) increasing the concentration of donors and acceptors in the reaction, (ii) lowering water activity of the reaction, (iii) shifting the reaction equilibrium to the end product direction by the product elimination in the medium, and (iv) altering the synthesis conditions [60,65].

β-Galactosidases come from different sources, such as *Aspergillus oryzae*, *Sterigmatomyces elviae*, *Bifidobacteria*, and *Lactobacilli*. Different sources of β-galactosidases cause various types of GOS that differ in the amount, DP, and glycosidic linkages [66,67,68,69]. Various sources of β-galactosidases need different conditions for optimizing GOS production. For example, fungal and bacterial, as well as yeast sources, require acidic and neutral pH, respectively. Furthermore, high temperature necessitates for thermophilic sources. These conditions have been optimized in various studies [66,70,71,72,73].

For GOS bio-catalysis, the whole cell or just the free form of β-galactosidase can be used. The recombinant form of this enzyme is also available. The whole cell is exploited when the β-galactosidase isolation process is uneconomical [74]. The utilization of the whole cell is also much cheaper due to co-factors that naturally exist in the cell and cell membrane [75,76], but it is not very crucial for GOS synthesis because β-galactosidase uses metal ions as co-factors.

There are some by-products, such as glucose and galactose, which do not have prebiotic effects and may decrease GOS synthesis yield. When the whole cell is used, these by-products can be removed by other metabolic processes. For instance, *Sirobasidium magnum*, *S. elviae*, and *Rasopone minuta* consume glucose as a carbon source when cultured on lactose medium for GOS synthesis [77,78,79,80,81]. As another example, galactose can induce the expression of β-galactosidase, and glucose is utilized as a carbon source in yeast cells [82]. However, some metabolic end products, including ethanol, lactic acid, and acetic acid, are produced, when viable whole cells are used, which can affect GOS production. Therefore, other methods are required to remove these metabolic products. Apart from the interference of metabolic end products with GOS production, the temperature is another unfavorable factor when using the whole cell. Temperature often increases the yield of GOS synthesis, which is undesirable and even fatal for non-thermophilic cells. In some studies, non-viable and resting cells are exploited. These kinds of cells do not have the drawbacks of viable cells, and their GOS production yields are much higher [57,66,83].

Recombinant β-galactosidases have more advantages than native β-galactosidases, such as high production yield, easy purification, and improved enzyme stability, as well as an activity through molecular approaches [84]. *Escherichia coli* and *Bacillus subtilis* are mostly used for producing recombinant β-galactosidases. *E. coli* has some disadvantages, such as endotoxins production, difficulty in disulfide bonds expression, and acetate formation, which has toxic effects [85,86]. In contrast, the engineered *B. subtilis* does not produce any endo- or exo-toxins. But this bacterium has also some disadvantages, including producing proteases in high quantities (which are able to degrade proteins) and plasmid instability [86,87].

Some yeasts, such as *S. cerevisiae* and *Pichiapastoris*, have been used for producing recombinant forms of β-galactosidase. Yeast has some advantages as compared to bacteria, including (i) higher range of productivity, (ii) disulfide bond production, and (iii) better protein folding [86,88,89].

## 5. Prebiotics Mechanisms for Alteration of Gut Microbiota

By the provision of energy sources for gut microbiota, prebiotics are able to modulate the composition and the function of these microorganisms [90]. Distant bacterial species in phylogeny share their skills to consume a specific prebiotic regularly [24]. It has also been recently reported by a functional metagenomics technique. In this method, genes from a human microbiota metagenomic library are identified for the breakdown of several prebiotics in a heterologous host, such as *E. Coli* [91]. Clones from various species, such as *Actinobacteria*, *Bacteroidetes*, and *Firmicutes*, can ferment FOS, GOS, and xylooligosaccharides (XOS). In contrast, some other studies report that specific species can degrade a given prebiotic. Fermentation of starch [92,93] and fructans [94] by *Bifidobacterium* sp. are examples in this regard. Another important factor for distinguishing species that are capable of fermenting a specific prebiotic is their chain length. For example, inulin (with DP of ≤60) can be fermented only by a few species, whereas a large number of microorganisms are able to degrade FOS (with DP of ≤10) [26].

Sometimes, a by-product of a complex prebiotic’s fermentation is a substrate for another microorganism, called cross-feeding [92,95]. For example, *Ruminococcus bromii* can degrade resistant starches, and several species can utilize the fermentation products of this reaction [31]. At the same time, some products may have antagonistic effects on other species.

Prebiotics are also able to modify the environment of the gut. As mentioned before, fermentation products of prebiotics are mostly acids, which decrease the gut pH. It has been shown that one unit alteration in the gut pH from 6.5 to 5.5 can contribute to a change in the composition and population of the gut microbiota [96,97]. The pH alteration can change the population of acid-sensitive species, such as *Bacteroids*, and promote butyrate formation by *Firmicutes*. This process is called butyrogenic effect [96].

## 6. Prebiotics Mechanisms for Health Maintenance and Protection against Disorders

**Figure 2:**
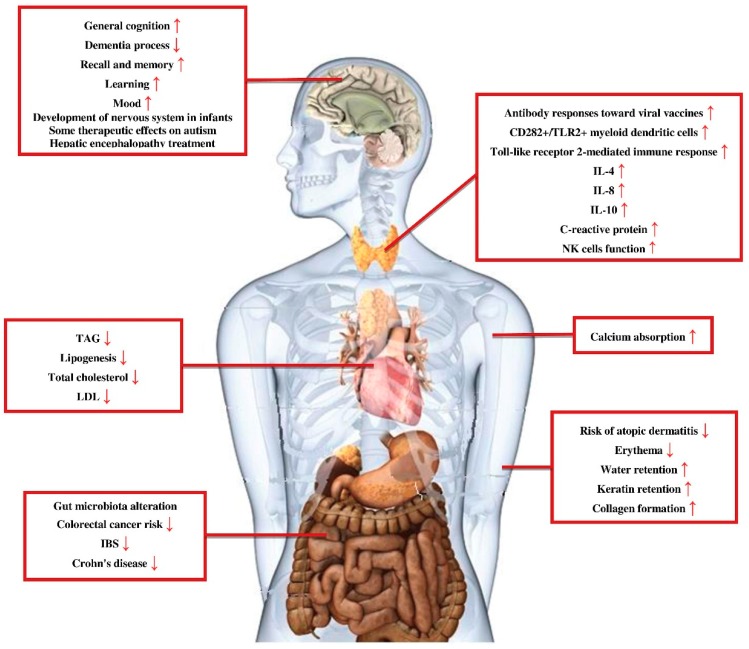
Prebiotics effects for health maintenance and protection against disorders. Prebiotics not only have protective effects on the gastrointestinal system but also on other parts of the body, such as the central nervous system, immune system, and cardiovascular system. TAG: triacylglycerol; LDL: low-density lipoprotein; IBS: irritable bowel syndrome; IL-4: interleukin 4; IL-8: interleukin 8; IL-10: interleukin 10; NK cells function: natural killer cells function.

As it was mentioned earlier, the products of prebiotics degradation are mainly SCFAs. These molecules are small enough to diffuse through gut enterocytes and enter blood circulation. Therefore, prebiotics are able to affect not only the gastrointestinal track but also other distant site organs and systems [18] (Figure 2).

### 6.1. Prebiotics and Gastrointestinal Disorders

#### 6.1.1. Irritable Bowel Syndrome and Crohn’s Disease

There are a few studies about the effects of prebiotics on irritable bowel syndrome (IBS) and Crohn’s disease. IBS is a gastrointestinal syndrome characterized by chronic abdominal pain and altered bowel habits in the absence of any organic cause. Crohn’s disease is a type of chronic, relapsing inflammatory bowel disease (IBD), which can involve any part of the gastrointestinal tract from the mouth to the anus. It has been reported that in both IBS and Crohn’s disease, the *Bifidobacteria* and *Faecalibacterium prausnitzii* population along with *Bacteroides* to *Firmicutes* ratio were decreased [29,98].

A double-blind cross-over study demonstrated that the administration of oligofructose at the dose of 6 g/day for 4 weeks had no therapeutic effects on patients suffering from IBS [99]. Similarly, another randomized, double-blind, placebo-controlled trial published in 2000 implicated that 20 g/day FOS supplementation failed to improve IBS [100]. In contrast, two more recent randomized, double-blind, clinical trials have shown IBS symptoms improvement after consuming 5 g/day FOS for 6 weeks [101] or 3.5 g/day GOS for 12 weeks [102].

A group study in 2006 reported that supplementation with 15 g/day FOS for 3 weeks elevated *Bifidobacteria* population in the feces and improved Crohn’s disease [103]. However, the other randomized, double-blind, and placebo-controlled trials demonstrated no clinical benefits after administrating 15 g/day FOS in patients with active Crohn’s disease [104] and 20 g/day oligofructose-enriched inulin in patients with inactive or mild-to-moderately active Crohn’s disease [105] for a duration of 4 weeks.

#### 6.1.2. Colorectal Cancer

Colorectal cancer, ranked as the third most common malignancy worldwide, is a multi-step disease from genetic mutation to adenomatous polyps, which then leads to invasive and metastatic cancer [106]. It has been demonstrated that prebiotics fermentation products, such as butyrate, could have protective effects against the risk of colorectal cancer, as well as its progression, via inducing apoptosis [106,107,108]. In addition, a clinical trial demonstrated that symbiotic therapy (*Lactobacillus rhamnosus* and *Bifidobacterium Lactis* plus inulin) could reduce the risk of colorectal cancer by reducing the proliferation rate in colorectal, inducing colonic cells necrosis, which leads to improving the integrity and function of epithelial barrier [106,109,110].

#### 6.1.3. Necrotizing Enterocolitis

Necrotizing enterocolitis (NEC) is a gastrointestinal emergency condition primarily in premature neonates, in which portions of the bowel undergo necrosis. It can lead to high morbidity and mortality rates [111]. Since prebiotics, such as FOS and GOS, can stimulate the growth of gut microbiota (e.g., *Bifidobacteria*) and reduce the pathogenic bacteria in preterm infants [112,113,114], it is claimed that they can prevent NEC [111]. Moreover, SCFAs can improve feeding tolerance by enhancing both gastric emptying and bowel motility [115,116,117]. A meta-analysis of four randomized controlled trials showed that FOS, GOS or their mixture could elevate the concentration of fecal *Bifidobacteria*, but had no significant effect on risk reduction and progression of NEC [118] (Table 1). Therefore, more clinical trials need to be done to elucidate the definite effects of prebiotics on NEC.

### 6.2. Prebiotics and the Immune System

Consuming prebiotics can improve immunity functions by increasing the population of protective microorganisms. Animal and human studies have shown that prebiotics can decrease the population of harmful bacteria by *Lactobacilli* and *Bifidobacteria* [12,121,122,123,124]. For example, mannose can reduce colonization of pathogens by promoting mannose adhesion to *Salmonella*. Mannose binds to *Salmonella* via type 1 fimbriae (finger-like projections) [125]. In addition, pathogens are not able to bind to the epithelium in the presence of OSCs. Prebiotics can also induce the expression of immunity molecules, especially cytokines (Table 2).

Interestingly, maternal prebiotics metabolites are able to cross the placenta and can affect the development of the fetal immune system [12,126]. In 2010, Fugiwara et al. [127] reported that FOS administration in a pregnant mouse model modified offspring microbiota, and consequently, their skin inflammation was attenuated. In contrast, Shadid et al. [128] in a placebo-controlled, randomized, and double-blinded study demonstrated that bifidogenic effects of prebiotics supplementation in humans could not be transferred to the next generation. The details of well-known prebiotic effects on the immune systems are discussed below:

*I-*  *Oligofructose and inulin mixture*: The mixture of oligofructans and inulin can improve antibody responses toward viral vaccines, such as influenza and measles [129].
*II-*  *FOS:* Studies have shown the improvement of antibody response to influenza vaccine following FOS consumption. Moreover, the side effects of the influenza vaccine are reduced [130,131]. Diarrhea-associated fever in infants is also reduced by this category of prebiotics. Apart from these, it can decrease the use of antibiotics, duration of disease, and the incidence of febrile seizures in infants [132,133].
  β(2→1) fructans can up-regulate the level of interleukin 4 (IL-4) in serum, CD282+/TLR2+ myeloid dendritic cells, and a toll-like receptor 2-mediated immune response in healthy volunteers [134]. In contrast, another study demonstrated that the salivary immunoglobulin A (IgA), immune cells in serum, and activation and proliferation of T cells and natural killer (NK) cells were not changed after consuming β(2→1) fructans [135]. It has been noted that FOS reduces the risk of some immune diseases in infants, such as atopic dermatitis [136,137]. This type of prebiotic decreases the expression of IL-6 and phagocytosis in monocytes and granulocytes [138].
*III-*  *GOS*: Studies showed that GOS increased the blood level of interleukin 8 (IL-8), interleukin 10 (IL-10), and C-reactive protein in adults, but decreased IL-1β. It has been found that the function of NK cells improves by consuming GOS [139,140]. In infants, GOS reduces the risk of atopic dermatitis and eczema [136,137,141].
*IV-*  *AOS (acidic oligosaccharides)*: The possibility of atopic dermatitis is reduced by AOS in low-risk infants [136].

### 6.3. Prebiotics and the Nervous System

The gastrointestinal tract is connected to the central nervous system through the “gut-brain axis” [142]. For instance, administration of prebiotics in piglets decreases the gray matter in order to improve neural pruning [143]. But the regulatory effects of prebiotics on the human brain have not been completely defined. Gut microbiota affects the brain through three routes, including neural, endocrine, and immune pathways [142,144,145].

*I-*  *Neural Pathway*: The products of prebiotics fermentation can affect the brain by the vagus nerve [146]. Some prebiotics, such as FOS and GOS, have regulatory effects on brain-derived neurotrophic factors, neurotransmitters (e.g., d-serine), and synaptic proteins (e.g., synaptophysin and N-methyl-D-aspartate or NMDA receptor subunits) [147,148].
*II-*  *Endocrine Pathway*: Hypothalamic-pituitary-adrenal axis is a neuroendocrine pathway. The microbiome growth in mice can induce corticosterone and adrenocorticotropic hormone in an appropriate way [149]. In addition, prebiotics act as a regulator of other hormones, such as plasma peptide YY [147].
*III-*  *Immune Pathway*: As discussed before, prebiotics can affect different aspects of the immune system. Beside neurological functions, prebiotics are also capable of influencing mood, memory, learning, and some psychiatry disorders by changing the activity and/or composition of gut microbiota [145] (Table 3).
*IV-*  *Mood*: Stress hormones are able to affect anxiety-related behaviors [150,151]. It was demonstrated that the level of stress hormones (adrenocorticotropic hormone (ACTH) and corticosterone) increased in germ-free mice following exposure to controlled stress. After administrating *Bifidobacterium infantis*, corticosterone and ACTH reached normal levels [149].
*V-*  *Memory, concentration, and learning*: Recently, a number of studies have shown the relation between memory and administration of fermentable compounds in both animals and humans [152]. Investigations on a different kind of prebiotics have implicated memory improvement in middle-aged adults [153,154]. Some prebiotics, such as arabinoxylan and arabinose, can enhance general cognition and attenuate the accumulation process of dementia-related glial fibrillary acidic protein in mice [155]. Prebiotics may be more efficient in preserving recall and learning rather than the development process.
  In 2015, a randomized, double-blind, and placebo-controlled study was performed to examine the effects of FOS and GOS daily consumption for three weeks on the level of salivary cortisol and emotional alteration regarding this hormone. FOS had no significant effect, but 5.5 g GOS intake increased the level of cortisol in saliva and enhanced the concentration in adults [156]. A randomized, double-blind, placebo-controlled trial demonstrated that administration of non-starch polysaccharides (3.6 g per day) for twelve weeks enhanced recall and memory processes in the middle-aged adult [153,154]. In contrast, the mixture of FOS, GOS, and AOS could not enhance the development of the nervous system in preterm infants after 24 months [157]. In two other clinical investigations, Smith et al. observed that administration of inulin-enriched oligofructose might enhance mood, recognition, immediate memory, and recall (after 4 hours). However, this prebiotic failed to recover long-term memory (after 43 days) [158,159].
  In another study, administration of polydextrose and GOS mixture decreased anxiety-like behavior in male piglets and promoted positive social interactions in rats [143,160]. Furthermore, the consumption of this mixture boosted their cognition memory [160,161].
*VI-*  *Autism*: 70% of people with autism are suffering from concomitant gastrointestinal disorders compared to 9% of healthy individuals. Chronic constipation (and other diseases as a result of constipation), abdominal pain with or without diarrhea, gastroesophageal reflux disease, abdominal bloating, disaccharide deficiencies, gastrointestinal tract inflammation, and enteric nervous system abnormalities are examples of gastrointestinal symptoms and signs that are reported for patients with autism spectrum disorders [162]. The severity of autism is shown to be correlated to higher gastrointestinal disorders [163]. Interestingly, a review article published in 2016 confirmed these statements [164].
  The composition of gut microbiota is changed in patients with autism disorders. Some studies have shown high levels of Clostridium and depleted *Bifidobacterium* in feces. In children with autism, gut metabolites are different from healthy individuals. For example, the amount of SCFAs in children with autism is lower than healthy ones [163,165]. Various prebiotics, such as wheat fiber, may have therapeutic effects on patients with autism by decreasing the population of *Clostridium perfringens* and increasing the rate of *Bifidobacteria* [166].
  Catecholamines, which are a category of neurotransmitters, are increased in individuals with autism. These neurotransmitters are produced by tyrosine hydroxylase. An in vitro study in a rat adrenal medulla cell line demonstrated that SCFAs, the products of prebiotic fermentation, could induce the expression of tyrosine hydroxylase [167]. However, further investigations are required to understand which prebiotics have therapeutic effects on human autism.
*VII-*  *Hepatic encephalopathy*: Hepatic encephalopathy happens when the liver does not function properly. The main reason for hepatic encephalopathy is the increases in the level of blood ammonia. This condition causes numerous psychiatric and neurologic complications, including personality, speech, and movement disorders, as well as cognition impairment, and may eventually result in coma and death.
  In 1966, it was demonstrated that lactulose could effectively treat hepatic encephalopathy by decreasing the level of ammonia in the gut. Lactulose can improve the life quality of people suffering from hepatic encephalopathy. This prebiotic also has preventive effects on hepatic encephalopathy [143,168,169,170]. Lactulose exerts its beneficial effects on hepatic encephalopathy through different pathways. First, the product of lactulose fermentation is lactic acid, which is able to reduce the colonic lumen pH by releasing H^+^. The ammonia in the gut reacts with proton and produces ammonium. This conversion develops a concentration gradient that increases the amount of ammonia reuptake from the blood into the gastrointestinal tract [171]. Second, in the presence of lactulose in the gastrointestinal tract, the bacteria utilize the energy of lactulose fermentation instead of the conversion of amino acids to ammonia energy. Third, lactulose can inhibit glutaminase and prevent the production of ammonia from glutamine [143]. Finally, lactulose shortens the colonic transit time. Thus, it can reduce the level of ammonia in the gastrointestinal tract. Other compounds, such as lactitol, may also be as effective as lactulose in the treatment of hepatic encephalopathy. Interestingly, the side effects of lactitol are much fewer than lactulose (e.g., flatulence and nausea) [172,173,174].

### 6.4. Prebiotics and Skin

As mentioned in the previous sections, the consumption of prebiotics was shown to decrease the risk of development, as well as the severity of allergic skin diseases, such as atopic dermatitis [136,137]. In hairless mice exposed to the UV, the consumption of GOS for 12 weeks enhanced water retention and also prevented the development of erythema [175]. On the other hand, GOS can improve skin barrier by increasing dermal expression of cell adhesion and matrix formation markers (e.g., CD44 and collagen type 1). Upon metabolizing aromatic amino acids by gut microbes, some compounds, such as phenols, may be produced. These compounds are transferred into the skin. Phenols, such as p-cresol, may be toxic for patients with underlying kidney diseases [176]. In women, consumption of GOS with or without probiotics, such as *Bifidobacterium breve*, can abolish the reduction of water and keratin caused by phenols [177,178,179,180] (Table 4).

### 6.5. Prebiotics and Cardiovascular System

According to the statistics, 30% of the deaths in the United States in 2013 were caused by cardiovascular diseases (CVD). The main reason for this growing trend is the alteration of people’s lifestyles and eating habits [181]. Therefore, many researchers have studied the influence of fibers and prebiotics consumption on CVD. However, the direct beneficial functions of prebiotics in this regard have not been demonstrated yet. In this section, we summarized some of the indirect effects of prebiotics on CVD.

Prebiotics are able to lower the risk of CVD by reducing the inflammatory elements. Several investigations demonstrated an improvement in the lipid profile by consuming prebiotics. In a randomized, double-blind, and placebo-controlled crossover clinical trial, Letexier et al. [182] treated healthy individuals with 10 g/day inulin for three weeks. They observed that this regimen decreased blood triacylglycerol (TAG) and liver lipogenesis, but it had no statistically significant effect on the cholesterol level.

In line with these findings, in a randomized and double-blinded cross-over trial, Russo et al. [183] demonstrated that the consumption of inulin-enriched pasta with a formulation of 86% semolina, 11% inulin, and 3% durum wheat vital gluten decreased both TAG and lipogenesis in healthy individuals, rather than cholesterol level. In contrast, Frochen and Beylot [184] reported that the consumption of 10 g/day inulin-type fructans for six months had no significant effects on lipogenesis in the liver of healthy subjects.

To assess the effects of oral L-rhamnose and lactulose on lipid profile in a partially randomized crossover study, Vogt et al. [185] administered 25 g/day of these two prebiotics for four weeks in healthy individuals. They observed a significant reduction in the synthesis and level of TAG but not cholesterol. Opposed to that, the results of another investigation in 1991 suggested that lactulose increased blood cholesterol (up to 10%) and B-apolipoprotein (up to 19%) [186].

In a double-blind, randomized, placebo-controlled, crossover study on overweight subjects with ≥3 risk factors of metabolic syndrome, Bimuno® Galacto-oligosaccharides (B-GOS) administration for 12 weeks decreased circulating cholesterol, TAG, and total:HDL (high-density lipoprotein) cholesterol ratio [187]. However, in the elderly, this prebiotic had no significant effect on the total:HDL cholesterol ratio [139]. The effect of β-glucan intake on lipid profile was measured in a meta-analysis study (from 1990 through Dec. 2009). It was implicated that β-glucan consumption could reduce the level of total cholesterol and LDL [188]. Finally, a meta-analysis of relevant randomized controlled clinical trials published between 1995 and 2005 implicated that FOS could reduce TAG level with an average rate of 7.5% [189].

Paradoxically, prebiotics may have a detrimental effect on lipid profile through producing some SCFAs, such as acetate. Acetate can be converted to acetyl-CoA, which is a substrate to synthesize fatty acids in hepatocytes [190]. This can justify the increase in the blood concentration of cholesterol and triglycerides after rectal infusion of acetate [191]. However, some other SCFAs, such as propionate and butyrate, may improve lipid profile. Propionate can inhibit lipid synthesis from acetate [192]. Therefore, prebiotics, such as FOS and L-rhamnose, may have lipogenic effects by producing acetate, butyrate, and propionate [14,193]. Hence, it is crucial to determine the end products of prebiotics to select the appropriate one for this purpose. Although prebiotics are claimed to be beneficial for obesity-related diseases, such as fatty liver disease, particularly, non-alcoholic fatty liver issue in one study [194], there is at least another clinical trial that refuted this opinion [195] (Table 5).

### 6.6. Prebiotics and Calcium Absorption

Statistics have shown that more than 28 million people in the United States have osteoporosis or low bone mass, and in European Union, one out of eight citizens over 50 years old have spinal fracture each year [196]. There are clinical trials on the impact of prebiotics dietary fibers on the absorption of minerals, such as calcium, but the results are conflicting. Some studies have shown that consumption of lactulose, TOS or inulin + oligofructose in doses ranged between 5 to 20 g/day significantly absorb calcium absorption. In contrast, such a phenomenon is not observed for GOS or FOS (Table 6) [197].

## 7. Prebiotics Safety

Prebiotics are assumed to lack life-threatening or severe side effects. Intestinal enzymes cannot break down oligosaccharides and polysaccharides. They are transported to the colon to be fermented by the gut microbiota. Therefore, the side effects of prebiotics are mostly the result of their osmotic functions. In this regard, osmotic diarrhea, bloating, cramping, and flatulence could be experienced in prebiotic recipients. The prebiotics chain length is an influential parameter for the development of their side effects. Interestingly, prebiotics with shorter chain length may have more side effects. The possible explanation for this phenomenon is that shorter inulin molecules are metabolized primarily in the proximal colon and are more rapidly fermented; whereas, longer chain ones are fermented later and slower in the distal colon. Beside chain length, the prebiotic dose can affect its safety profile. For example, low (2.5–10 g/day) and high (40–50 g/day) doses of prebiotics can cause flatulence and osmotic diarrhea, respectively. Noting that, a daily dose of 2.5–10 g prebiotics is required to exert their beneficial functions on human health. This means that prebiotics within their therapeutic doses can cause mild to moderate side effects. Most products of prebiotics in the market have doses of 1.5–5 g per portion [206].

As potential alternatives or adjunctive therapies (synbiotics) to probiotics [207], prebiotics may have similar safety concerns. The major safety issue of probiotics includes the risk of bacteremia, sepsis or endocarditis, especially in patients with prominent immuno-deficiency (e.g., HIV, cancer, transplant), severe malnutrition or incompetent intestinal epithelial barrier (e.g., severe diarrhea, NEC) [208]. It is noteworthy that these potential complications have not been considered or at least reported in relevant clinical studies exclusively for prebiotics.

## 8. Conclusions

Prebiotics exert a remarkable influence on human health, which makes them alluring attractive agents to improve the quality of human life against cancer, vascular diseases, obesity, and mental disorders. There are many studies on the positive effects of prebiotics on human health; however, accurately designed long-term clinical trials and genomics investigations are needed to confirm the health claims.

By determining the fundamental mechanisms of prebiotics, scientists would be able to formulate enhanced food supplements to ameliorate human health. The ability to normalize the composition of the gut microbiota by prebiotic dietary substances is an appealing procedure in the control and healing of some foremost disorders. In other words, the gut microbiota, as a major body organ, can be fed properly with prebiotics to become stronger and healthier, which, in turn, can impact the overall health.

Considering the diversity of the gut microbiota in various populations and countries, and even in different individuals, based on the variety of dietary regimens, developing effective and diverse probiotics for the modification of the microbiota hemostasis seems not to be very feasible. On the other hand, prebiotics seem to be a more convenient option in this regard, especially due to a much easier production and formulation process, as well as lack of need for cold chain in transportation and storage. The negligible side effects of prebiotics are also an important advantage.

Therefore, designing particular, population-specific prebiotics with regard to the resident gut microbiota specific to each community may ultimately contribute to the reduction of certain disorders in each society as a standardized approach. This concept provides the potential to stop the huge prebiotic controversies and can be recommended in future guidelines from the FAO and/or the WHO on prebiotics.

## Figures and Tables

**Table 1:** Studies showing the effect of prebiotics on the gastrointestinal tract.

Prebiotic	Dose	Subjects	Main Results	Reference
**FOS**	6 g/day for 4 weeks	Patients with IBS	No therapeutic effect.	[99]
	20 g/day for 12 weeks	Patients with IBS	No therapeutic effect.	[100]
	5 g/day for 6 weeks	Patients with IBS	Improvement in IBS syndromes.	[102]
	15 g/day for 3 weeks	Patients with active ileocolonic Crohn’s disease	Crohn’s disease improvement.	[103]
	15 g/day for 4 weeks	Patients with Crohn’s disease	No clinical improvement in Crohn’s disease.	[104]
**GOS**	3.5 g/day for 12 weeks	Patients with IBS	Improvement in IBS syndromes.	[102]
**Mixture of FOS and GOS**	0.8 g/dL of a mixture of GOS and FOS, ratio 9:1 for 30 days	Healthy newborns	Improvement in gastric emptying and bowel motility.	[115]
	0.8 g/dL of a mixture of GOS and FOS, ratio 9:1 for 15 days	Healthy newborns	Improvement in gastric emptying and bowel motility.	[116]
**Inulin-enriched FOS**	20 g/day for 4 weeks	Patients with inactive and mild to moderately active Crohn’s disease	No clinical Improvement in Crohn’s disease.	[105]
	Raftilose^®^ Synergy 1 + *Bifidobacterium lactis* Bb12, *Lactobacillus rhamnosus* GG	HT29 or CaCo-2 cells	Cell growth inhibition. As a result, this mixture can decrease the progression of colorectal cancer.	[119]
	Different doses	Rats with colon carcinogen	Long-chain inulin effects are dose-dependent on colorectal cancer.	[120]
	Synergy 1 + *Bifidobacterium lactis* Bb12, *Lactobacillus rhamnosus* GG	Colon cancer patients and polypectomized patients	Decrease in the progression of colorectal cancer.	[110]
**Lactose**	25 g daily for 15 days	Lactose malabsorbers	Improvement in lactose digestion.	[117]

FOS: Fructo-oligosaccharides; IBS: irritable bowel syndrome; and GOS: Galacto-oligosaccharides.

**Table 2:** Studies showing the effect of prebiotics on the immune system.

Prebiotic	Dose	Subjects	Main Results	Reference
**FOS**	8 oz/day of an experimental formula containing FOS for 183 days	Adults aged 65 and older	Antibody responses toward viral vaccines improved. Hospitalization due to influenza and side effects of influenza vaccines decreased.	[130]
	8 g/day Orafti^®^ Synergy1 for 8 weeks	Adults aged 45–63 years	Immune responses toward influenza vaccines improved.	[135]
	0.55 g FOS per 15 g of cereal for 6 months	Non-breast-feeding infants aged 4–24 months	Diarrhea associated fever, febrile seizure incident, antibiotics usage, and duration of infectious disease decreased.	[133]
	3 × 5 g/day FOS consisted of two 28 day treatments separated by a 14-day washout	Healthy volunteers	IL-4 in serum, CD282+/TLR2+ myeloid dendritic cells, and toll-like receptor 2-mediated immune response were up-regulated.	[134]
	Not exactly defined	Infants	Risk of some immune diseases, such as atopic dermatitis, reduced.	[136,137]
	2 × 4 g/day for 3 weeks	Elderly nursing home patients	IL-6 expression and phagocytosis in monocytes and granulocytes decreased.	[138]
	8 g/day Orafti^®^ Synergy1 for 4 weeks	Adults aged 45–65 years	Salivary IgA, immune cells in serum, activation, and proliferation of T and NK cell not changed.	[131]
**GOS**	5.5 g/day for 10 weeks	Elderly subjects	Phagocytosis, NK cell activity, and IL-10 (an anti-inflammatory cytokine) level increased. Pro-inflammatory cytokines, such as IL-6, IL-1β, and tumor necrosis factor-α, levels decreased.	[139]
	5.5 g/day consisted of two 10 weeks of treatment separated by 4 weeks of washout	Elderly subjects	IL-10, IL-8, C-reactive protein, and NK cell activity elevated. IL-1β level decreased.	[140]
	Not exactly defined	Infants	Risk of some immune diseases, such as atopic dermatitis, reduced.	[136]
	0.8 g/100 mL	Infants		[137]
	0.8 g/day for 6 months	Newborn infants		[141]
**AOS**	Not exactly defined	Infants	Atopic dermatitis in low-risk infants reduced.	[136]
**Oligofructose and inulin mixture**	Oligofructose (70%) and inulin (30%) with a concentration of 1 g per 25 g of dry weight cereal during 4 weeks prior to measles vaccination	Infants aged 7–9 months	Antibody responses toward viral vaccines improved.	[129]

FOS: Fructo-oligosaccharides; IBS: irritable bowel syndrome; GOS: Galacto-oligosaccharides; AOS: acidic oligosaccharides; NK cell: natural killer cell; IL-4: interleukin 4; IL-10: interleukin 10, IL-8: interleukin 8; and IL-6: interleukin 6.

**Table 3:** Studies showing the effect of prebiotics on the nervous system.

Prebiotic	Dose	Subjects	Main Results	Reference
**Non-starch polysaccharides (NSPs)**	4 g of NSPs (Ambrotose^®^)	Middle-aged healthy adults	Recognition and working memory performance improved.	[153]
	3.6 g/day for 12 weeks	Middle-aged healthy adults	Cognitive function and well-being optimized.	[154]
**Mixture of FOS, GOS, and AOS**	Supplementation between day 3 and 30 of life, and the results measured during 24 months	Preterm infants	Neurodevelopment did not improve significantly.	[157]
**Inulin-enriched oligofructose**	5 g, the results measured after 4 h	19–30 years old healthy individuals	Mood, recognition, immediate memory, and recall enhanced.	[158]
	10 g/day of Synergy^®^ 1, the results measured after 43 days	19–64 years old healthy individuals	Long-term memory did not change significantly.	[159]
**Mixture of GOS and polydextrose**	2.4 and 7 g/L of polydextrose and GOS	Male piglets	They may have neurodevelopment effect in human infants.	[143]
	7 g/kg prebiotics mixture	Rats	Memory and social behaviors improved, and anxiety-like behaviors reduced.	[160]
	15 g/kg prebiotics mixture	Mice		
**Water extract of *Triticum aestivum* composed of arabinoxylan, β-glucan, and arabinose**	-	Rats	Arabinoxylan, β-glucan, and arabinose had preserved cognition effects against vascular dementia.	[155]
**GOS**	5.5 g/day for 3 weeks	18–45 years old healthy volunteers	Salivary cortisol awakening response was decreased, attentional vigilance to negative versus positive information reduced, and the concentration improved.	[156]
**Lactulose**	Lactoferrin (0.6 g/L) and Milk fat globule membrane (MFGM) (5.0 g/L)	Male piglets	Lactulose appeared to have neurodevelopment effect in human infants.	[143]
	Duphalac^®^ 90–150 mL/d	Patients with chronic portal-systemic encephalopathy (PSE)	Blood ammonia levels decreased.	[168]
	30–60 mL of lactulose in 2 or 3 divided doses for 3 months	Patients with cirrhosis	Cognitive function and health-related quality of life improved.	[169]
	Meta-analysis	Patients with subclinical hepatic encephalopathy	Lactulose had the most beneficial influence among prebiotics and probiotics.	[170]
	67 mg/day for long-term therapy (1 to 10 months)	Patients with chronic PSE	The lower intestinal tract was acidified, and lactulose had a beneficial effect on chronic PSE.	[171]

NSPs: non-starch polysaccharides; FOS: Fructo-oligosaccharides; GOS: Galacto-oligosaccharides; and AOS: acidic oligosaccharides.

**Table 4:** Studies showing the effect of prebiotics on the skin.

Prebiotic	Dose	Subjects	Main Results	Reference
**AOS**	Not exactly defined	Infants	Formula supplementation with a specific mixture of oligosaccharides was effective in preventing atopic dermatitis in low-risk infants.	[136]
**GOS**	Not exactly defined	Infants	Risk of some immune diseases, such as atopic dermatitis, reduced.	[136]
	0.8 g/100 mL	Infants		[137]
	0.8 g/day for 6 months	Newborn infants		[141]
**GOS with or without probiotics**	100 mg of GOS daily for 12 weeks	Hairless mice exposed to the UV	Water retention enhanced, and erythema reduced.	[175]
	600 mg of GOS for 4 weeks	Adult healthy women	Water and keratin reduction caused by phenols decreased.	[177]

**Table 5:** Studies showing the effect of prebiotics on the cardiovascular system.

Prebiotic	Dose	Subjects	Main Results	Reference
**Inulin-enriched pasta**	2-weeks run-in period, a baseline assessment, two 5-weeks study periods (11% inulin-enriched or control pasta)	Healthy individuals	HDL-cholesterol level elevated; total cholesterol/HDL-cholesterol ratio, triglycerides, and lipoprotein A levels reduced.	[183]
**Inulin**	10 g/day for 3 weeks	Healthy individuals	Hepatic lipogenesis and plasma triacylglycerol concentrations reduced.	[182]
**Mixture of inulin and oligofructose**	10 g/day for 6 months	Healthy individuals	Plasma triacylglycerol concentrations and hepatic lipogenesis were not changed. A non-significant decreasing trend in plasma total and low-density lipoprotein cholesterol levels were observed, and high-density lipoprotein cholesterol concentration increased.	[184]
**L-rhamnose**	25 g/day for 4 weeks	Healthy adults	Triacylglycerol (TAG) and net TAG-fatty acid (TAGFA) synthesis decreased.	[185]
**Lactulose**	25 g/day for 4 weeks	Healthy adults	Triacylglycerol (TAG) and net TAG-fatty acid (TAGFA) synthesis decreased.	[185]
	18–25 g/day for 2 weeks	Healthy individuals	Free fatty acid concentrations were reduced by increasing the absorbed acetate from the colon.	[186]
**GOS**	Administrating Bi2muno (B-GOS) for 2 six weeks	Overweight subjects with ≥3 risk factors of metabolic syndrome	Circulating cholesterol, TAG, and total:HDL cholesterol ratio decreased.	[187]

**Table 6:** Studies showing the effect of prebiotics on mineral absorption.

Prebiotic	Dose	Subjects	Main Results	Reference
**Inulin or oligofructose**	17 g of inulin or oligofructose and 7 g for three experimental periods of three days each.	Patients with conventional ileostomy because of ulcerative colitis	No significant effect on calcium, magnesium, zinc, and iron absorption.	[198]
**FOS or GOS**	15 g/day for 3 weeks	Healthy, nonanemic, male	No significant effect on calcium and iron absorption.	[199]
**Short chain FOS**	10 g/day for 5 weeks	Healthy, postmenopausal women	No significant effect on calcium absorption.	[200]
**FOS enriched milk**	5 g FOS/L with light breakfast	Healthy adults	No significant effect on calcium absorption.	[201]
**Lactulose**	5 or 10 g per day for two 9 days with 19-day washout in between	Post-menopausal women	Calcium absorption increased in a dose-response way.	[202]
**Trans-galacto-oligosaccharides**	20 g for two 9 days with 19-day washout in between	Post-menopausal women	Calcium absorption increased.	[203]
**A mixed short and long degree of polymerized inulin-type fructan product**	8 g/day for 8 weeks or 1 year		Calcium absorption increased significantly.	[204]
**The mixture of inulin + oligofructose**	8 g/day for two 3 weeks, separated by a 2-week washout period	Girls at or near menarche.	Calcium absorption increased.	[205]
